# Early postnatal low-protein nutrition, metabolic programming and the autonomic nervous system in adult life

**DOI:** 10.1186/1743-7075-9-80

**Published:** 2012-09-11

**Authors:** Júlio Cezar de Oliveira, Sabrina Grassiolli, Clarice Gravena, Paulo Cezar Freitas de Mathias

**Affiliations:** 1 Paulo Cezar de Freitas Mathias; Department of Cell Biology and Genetics Laboratory of Secretion Cell Biology, State University of Maringá, Block H67, room 19, State University of Maringá/UEM - Colombo Avenue 5970, 87020-900, Maringá, PR, Brazil; 2 Department of General Biology, State University of Ponta Grossa, General Carlos Cavalcanti Avenue 4847, 84030-900, Ponta Grossa, PR, Brazil

**Keywords:** Lactation, Metabolic programming, Insulin secretion, Pancreatic islets, Autonomic nervous system

## Abstract

Protein restriction during lactation has been used as a rat model of metabolic programming to study the impact of perinatal malnutrition on adult metabolism. In contrast to protein restriction during fetal life, protein restriction during lactation did not appear to cause either obesity or the hallmarks of metabolic syndrome, such as hyperinsulinemia, when individuals reached adulthood. However, protein restriction provokes body underweight and hypoinsulinemia. This review is focused on the regulation of insulin secretion and the influence of the autonomic nervous system (ANS) in adult rats that were protein-malnourished during lactation. The data available on the topic suggest that the perinatal phase of lactation, when insulted by protein deficit, imprints the adult metabolism and thereby alters the glycemic control. Although hypoinsulinemia programs adult rats to maintain normoglycemia, pancreatic β-cells are less sensitive to secretion stimuli, such as glucose and cholinergic agents. These pancreatic dysfunctions may be attributed to an imbalance of ANS activity recorded in adult rats that experienced maternal protein restriction.

## Introduction

The developmental origins of health and disease (DOHaD) hypothesis, which stipulates that adult metabolic disease may be programmed during the perinatal stage, has been tested in several experimental animal models. A large amount of evidence suggests that the etiology of obesity is not only related to food abundance but also to food restriction during early life [1-5]. There are numerous data showing that nutrient deprivation to the fetus increases the risk of developing metabolic disease in adult life [6,7]. Lactation, similarly to uterine life, is a very important phase for brain development, in rodent species in particular and, thus, constitutes another sensitive window during which nutritional insults can lead to the programming of adult metabolic disease [8,9]. Indeed, over nutrition during lactation provokes obesity and hyperinsulinemia, among other hallmarks of metabolic syndrome, whereas under nutrition permanently decreases body weight and causes metabolic changes. Because insulin plays a key role in the genesis of metabolic disease, this review will be dedicated to the effects of malnutrition during lactation on the regulation of insulin secretion via putative modifications of the autonomic nervous system (ANS).

### Autonomic nervous system and metabolism

One of the remarkable roles of the ANS is to help the central nervous system regulate the metabolism. Functioning as a bi-directional data conduit, afferent and efferent trunk nerves inform the brain regarding the peripheral metabolic status and return signals to the different tissues to maintain the metabolic homeostasis, respectively. The electrical stimulation of the ventromedial hypothalamic nucleus induces the immediate activation of hepatic glycogenolysis [10], whereas the stimulation of the lateral hypothalamus leads to a prompt enhancement of glycogen synthesis that allows an increase in blood glucose levels [11]. Both effects are mediated by the ANS and by the sympathetic and parasympathetic branches [12]. The hepatic vagus nerve senses glucose production in the liver and sends signals to the central nervous system, which integrates this message to control the metabolism [13]. Indeed, central and peripheral neural signals contribute to the control of the blood glucose concentration and to the maintenance of the body energy homeostasis. These neural mechanisms are thus pivotal to the regulation of body weight. The neural control of blood glucose levels also involved many endocrine factors, particularly those released by the pancreas and the adrenal medulla. During fasting and feeding sessions, the sympathetic and parasympathetic nervous system (PNS) activities adapt to maintain normoglycemia. A network of ANS neurons acting on target endocrine cells stimulates or inhibits the release of these hormones [14]. Postprandial hyperglycemia induces an increase in the PNS activity, which, throughout the vagal ends, potentiates glucose-induced insulin release from the pancreatic β-cells. This rise in the insulin levels rapidly provokes a fall in the blood glucose levels. However, the sympathetic nervous system, through the impulses of the splanchnic neurons, stimulates the adrenal medulla chromaffin cells to secrete catecholamines (mostly adrenaline) that increase the blood glucose concentration by hepatic glucose production and by blocking insulin secretion [15]. The electrical stimulation of the vagus or the splanchnic nerve alters the insulin or glucagon output from the pancreas, respectively [16]. The deterioration of these mechanisms thus might also be involved in the modulation of body weight [17-19].

**Figure 1:**
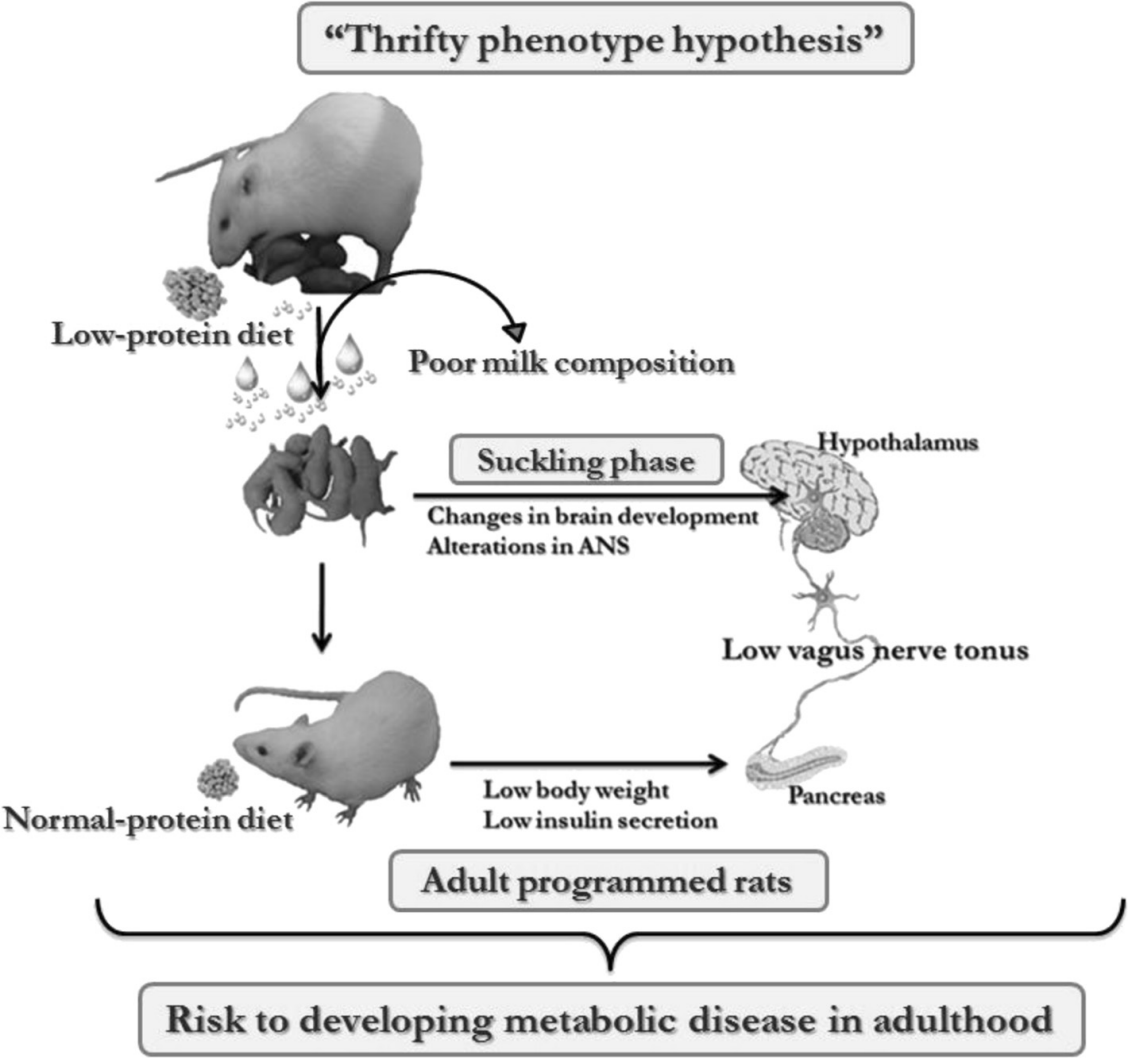
**Schematic model of metabolic programming resulting from a maternal low-protein diet during lactation, the “Thrifty phenotype hypothesis” and the development of metabolic disease in late life.** ANS, Autonomic nervous system.

Much attention has been paid to insulin over secretion, which is observed in obesity. An imbalance of ANS activity has been suggested as one cause, among others, of this pancreatic β-cell dysfunction. In obese humans and rodents, high vagus nerve activity has been observed, whereas reduced sympathetic tonus has been reported [20-23]. However, although less attention has been focused on the pathophysiology of the onset of body underweight, several studies have reported that the ANS activity is altered in under- or malnourished organisms [24,25]. After weaning, rats fed a chronically protein-deficient diet exhibited low activity of the vagus nerve, whereas high sympathetic activity was recorded, and these data were in agreement with a low insulin response to glucose [25]. In another study, pancreatic islets isolated from protein-restricted rats showed weak glucose and cholinergic insulin tropic responses [24], suggesting that the pancreatic β-cell dysfunction may be attributed to altered ANS activity in these underweight animals, as represented in schematic Figure 1.

Food abundance or restriction with regard to body weight control involves changes in metabolic homeostasis and ANS balance activity. Although the secretion of insulin by the pancreatic β-cells is increased in people who were overweight, it is diminished in people who were underweight. Changes in the ANS activity may constitute the mechanisms underlying the β-cell dysfunction: the high PNS tonus observed in obese individuals constantly potentiates insulin secretion, whereas the low activity reported in underweight individuals is associated with a weak cholinergic insulin tropic effect [26].

With the growing worldwide obesity epidemic, including huge populations in developing countries, such as China, India, Mexico and Brazil, the causes of this health and economic catastrophe have been increasingly studied [27-30]. It is well known that metabolic syndrome and obesity exhibit a high correlation with low or absent physical exercise practices and the consumption of calorie-rich diets in developing countries; however, although the inhabitants may actually experience a nutrition transition, high levels of overweight and obese individuals could not be justified solely by diet and physical inactivity, other hallmarks, such as metabolic programming by the under nutrition early in life and epigenetic modification could also be underlining the obesity onset.

### Under nutrition early in life and epigenetic modifications, association with metabolic diseases risk

In addition to the pathophysiological aspects that have emerged from studies on metabolic programming caused by environmental insults during fetal life [31,32], another interesting point that is relevant to this issue is the role of epigenetic changes in the increased risk of developing metabolic diseases, such as type 2 diabetes and obesity, later in life. Epigenetic mechanisms, such as DNA methylation and/or nucleoprotein acetylation/methylation, are crucial to the normal/physiological development of several tissues in mammals, and they involve several mechanisms to guarantee fluctuations of enzymes and other proteins that regulate the metabolism [33,34]. As previously reviewed, the intrauterine phase of development is particularly important for the genomic processes related to genes associated with metabolic pathways [34-36]. Therefore, this phase of life may be particularly important for nutritional disturbance. In humans who experienced the Dutch famine Winter in 1944-1945 and in rats that were deprived of food *in utero*, epigenetic modifications were detected in the insulin-like growth factor 2 (IGF2) and pancreatic and duodenal home box 1 (Pdx1), which are the major factors involved in pancreas development and pancreatic β-cell maturation [37,38]. Although it is known that the pancreas and the pancreatic β-cells develop/maturate during the embryonic phase [39], the postnatal life is also crucial for the maintenance processes that control the β-cell mass, such as proliferation, neogenesis and apoptosis [40]. Nevertheless, no data on metabolic programming as the result of protein-restricted diet during lactation only have yet been reported, and no direct association with epigenetic modifications has been observed; on the other hand, because stressor insults during the milk suckling phase can lead to disturbances in glucose metabolism, hypothalamic neurons, ANS activity and β-cell mass/function of the pancreatic β-cells in rodents, further studies are needed on this topic.

### Nutritional restriction to the fetus: a risk of obesity onset

Two decades ago, it was observed that low birth weight was related to adult chronic, non-transmissible diseases, such as type 2 diabetes, cardiovascular disease and obesity [2]. It has been speculated that a nutritional injury during perinatal growth, including uterine and early postnatal life, may contribute to adapting the adult metabolism toward nutritional restriction. However, if an abundant diet is offered to people who have been undernourished during the perinatal life, this opportunity induces a metabolic shift toward the storage of energy and high fat tissue accumulation, thus leading to high risks of the onset of metabolic/coronary diseases onset [1]. These observations led to the introduction of the term DOHaD (Developmental Origins of Health and Disease) previously known as the Barker thrifty phenotype hypothesis [41]. Currently, the concept of DOHaD is extended to any other insults during perinatal life, pregnancy and/or lactation, such as underweight, overweight, diabetic or hyperplasic mothers. This concept also includes any type of stressful situations that may predispose babies or pups to develop metabolic disorders when they reach adulthood [42-47].

### Food deficit in lactation does not cause obesity

Using perinatal under nutrition experimental models, it is important to score which hallmarks are presented by programmed organism, unless we can misunderstanding DOHaD concepts. It is known that restricting the food supply to a fetus may cause obesity, hyperinsulinemia and peripheral insulin resistance, among other clinical signals of metabolic syndrome, in adult life [2]. In contrast, over nutrition during lactation also induced obesity with the same metabolic abnormalities observed in the offspring from mothers undernourished during pregnancy [48,49], and food deficiency during the lactation period usually leads to underweight, not only in the young but also in the adults, even when dietetic recovery is available. Adult rats from mothers that experienced protein restriction during lactation show impairment of glycemic homeostasis. These programmed rats can no longer secrete as much insulin as the rising blood glucose levels demand [8]. However, the high insulin-sensitivity of their tissues is able to maintain normoglycemia [50]. Similar results were found in human populations that were poorly fed at the perinatal stage [51]. Pancreatic islets isolated from programmed lean adult rats show low glucose insulin tropic responses [52-54]. These results demonstrate that a protein-poor diet during lactation, in contrast to chronic protein malnutrition during the intrauterine and post-weaning phases, does not induce obesity and/or diabetes but rather reduces the pancreatic β-cell function. This adaptation allows the rat pancreas to release a low amount of insulin in response to altered metabolic demand. High insulin sensitivity may further impact the metabolism when the caloric intake increases, potentially contributing to future overweight followed by the hallmarks of metabolic syndrome, such as insulin resistance [55]. Consistent with this possibility, it has been reported that a 50% global food restriction during the last third of pregnancy and lactation, which are crucial periods for rodent brain development, led to modifications of the stress neuroendocrine systems, such as increased hypothalamus-pituitary-adrenal axis activity [56] and decreased sympathoadrenal system activity in adult male rats under resting conditions [57]. However, when a normal diet was offered, the adult male rats exhibited underweight rather than obesity, suggesting that under nutrition during lactation, at least in rodents, prevents individuals from becoming obese. In addition, it has been reported that prenatally undernourished rats nursed by control mothers exhibit a rapid catch-up growth and are more susceptible to developing obesity and features of metabolic syndrome [58]. In contrast, the same animals, if nursed by their own mothers, did not experience catch-up growth and exhibited only subtle metabolic alterations [59]. Taken together, these data indicate that lactation is a very sensitive period that is critical to programming the metabolism of adult rats and suggest that under nutrition during this period may protect rats from developing obesity. However, it remains to be determined whether this “protective” effect of under nutrition during lactation persists in the presence of a high caloric diet or physical inactivity.

### Sex dimorphism in metabolic programming

Importantly, some dysfunctions associated with metabolic programming are not similar between males and females. These dysfunctions are dependent not only on the window in which the nutritional disturbance (under/over nutrition) occurred but also on physiological differences between the genders [60-62]. As previously reported, glucose homeostasis, insulin sensitivity, pancreatic β-cell function and adipose tissue depots, among other metabolic hallmarks of adulthood, are changed by early maternal protein restriction [63-67]. These changes, including their magnitude, are expressed in a different manner both in female and male rats. Protein malnourishment during lactation causes greater insulin sensitivity in males than in females [63,67], and this is also observed when the protein restriction occurs during pregnancy and lactation, which leads to higher insulin sensitivity in males [66,67], while insulin resistance is observed in females [65]. However, contradicting data have also been reported [64]. Differences in glucose homeostasis impairments are observed dependent on gender in adult rats that were protein restricted prenatally; however, the period of nutritional insult is important to determine both the magnitude and the quality of the metabolic disturbance.

### Programmed metabolism and insulin secretion-coupling process

What are the mechanisms involved in the low glucose insulin tropic response observed in low protein-programmed lean rats? The pancreatic β-cells secrete insulin when stimulated mostly by glucose. However, several nutrients, such as amino acids, fatty acids, and their metabolites, can also stimulate cellular metabolism and increase ATP production [68,69]. ATP-sensitive potassium channels (K_ATP_) are inactivated by an increased ATP/ADP ratio, which provokes membrane depolarization and subsequently the activation of voltage-dependent calcium channels [70,71]. These ionic changes increase the intracellular calcium concentration, which is involved in the export of insulin to the bloodstream. However, experimental evidence indicates that glucose may also stimulate insulin secretion by alternative pathways involving K_ATP_ channels [72]. The use of adult rats that were protein-restricted during lactation allowed researchers to verify whether cell metabolism and cell calcium handling are involved in β-cell dysfunction. Although the results showed no change in either β-cell metabolism or calcium buffering, an impairment of cholinergic transduction was reported [73]. Surprisingly, diabetic rats transplanted with pancreatic islets from adult rats that were protein-restricted during lactation showed improvement of their fasting glycemic, which may indicate that changes in β-cell activity provoked by poor perinatal protein nutrition are not permanent and could open possibilities to new therapeutics to treat metabolic diseases [74].

### Programmed metabolism and insulin tropic effects of neurotransmitters

Insulin release is modulated by non-nutrient secretagogues, such as neurotransmitters, which enhance or inhibit glucose-stimulated insulin secretion. Pancreatic β-cells contain several receptors for neurotransmitters and Neuropeptide, such as adrenoceptors and cholinergic muscarinic receptors (mAChRs). These receptors are stimulated by efferent signals from the central nervous system, including the ANS, throughout their neural ends for pancreatic β-cells [75]. During blood glucose level oscillations, the β-cells receive inputs from the parasympathetic and sympathetic systems to participate in glycemic regulation. Overall, acetylcholine promotes the potentiation of glucose-induced insulin secretion, whereas noradrenaline and adrenaline inhibit this response [19]. Before and during food intake, vagus nerve activity is responsible for the rise in insulin blood levels. These effects are mediated by acetylcholine, which activates the mAChRs in the β-cell plasma membrane. Pancreatic islets isolated from rats that had been nursed by dams fed with a low-protein (4-8%) diet during the first 14 days of the lactation period showed a weak cholinergic response [53,73]. To date, five subtypes of receptors belonging to the muscarinic family (M_1_-M_5_) have been identified in neurons and other cell types [76,77]. Using the RT-PCR technique and different muscarinic antagonists, four subtypes (M_1_-M_4_) have been shown to be present in insulin-secreting cell lines [78], whereas other studies have shown that 2 muscarinic subtypes (M_1_ and M_3_), and in a lesser quantity, M_5_, are expressed in rat pancreatic islets [79]. Because pancreatic islets contain four different endocrine cell types, care must be taken when dealing with the mAChR subtypes of pancreatic β-cells. Functional studies of mAChR subtypes have revealed that M_1_ and particularly M_3_ are the receptors that are involved in the insulin tropic effect of acetylcholine [80,81]. Interestingly, it was reported that M_3_mAChR gene knockout mice are underweight, hypophagic and hypoinsulinemic [82], as are adult rats that were protein-restricted during lactation. The pancreatic islets from M_3_mAChR mice (-/-) showed a reduced secretory response to cholinergic agonists [83]. Although it has not been demonstrated, it is possible that metabolic programming induced by early protein malnutrition causes a reduction of M_3_mAChR in the pancreatic β-cell. Recently, in studies using transgenic mice in which the pancreatic β-cell M_3_mAChRs are chronically stimulated, an improvement of glycemic control has been observed, even when the mice received a high-fat diet [84]. These data may indicate that PNS tonus, intense or weak, can modulate β-cell function through muscarinic receptor activities. Three-month-old male adult offspring of mothers that received 4% protein during lactation exhibited low PNS activity. Fasting normoglycemia and hypoinsulinemia, glucose intolerance and low plasma glucose and insulin concentrations during an intravenous glucose tolerance test were observed in the adult offspring of malnourished mothers [53]. In these animals, acetylcholine or the blockage of the α_2_-adrenoreceptors increased the plasma glucose and insulin concentrations, whereas atropine or the stimulation of the α_2_-adrenoreceptors did not modify these parameters. In addition, islets from postnatally malnourished animals exhibited decreased insulin secretion in response to low glucose and carbachol. By contrast, changes in the sympathetic activity were not observed. Taken together, these data suggest that the hypoinsulinemia observed in the adult offspring of malnourished mothers might be caused by alterations in the regulation of glucose-induced insulin secretion via, in part, ANS modulation. However, other authors using maternal protein restriction to 8% protein during the entire lactation period concluded that the changes in the metabolism, such as hypoinsulinemia and low fat deposition, are due to the enhanced activity of the sympathoadrenal axis in 180-day-old rats [85]. The discrepancies between these studies might be because of the different regimens used or because the ages of the adult rats were different. In chronically protein-malnourished rats, a decrease in the PNS activity and a strong increase in the sympathetic nervous system activity were observed, as demonstrated by recordings obtained directly from the nerves of 90-day-old rats [25]. Recently, a low PNS activity was observed in the adult offspring of malnourished mothers, as directly recorded from the superior vagus nerve branch, whereas the firing rates of the sympathetic branch were unchanged [5]. Although the recorded electrical signal of vagus nerve activity is considered to be a combination of afferent and efferent signals, the firing rates observed in this study represent part of the electrical signal that travels from the brain to the thoracic and visceral organs, such as the pancreas [86]. The reduced cholinergic insulin tropic response of the pancreatic islets from metabolically programmed rats does not suggest an up-regulation effect due to the weak vagal activity, as expected. In contrast, studies indicate a low secretory sensitivity to the cholinergic stimulus in the β-cells from adult rats that were protein-malnourished during lactation [53,73,86]. The nature of the mAChR subfamilies that are implicated in the weak cholinergic response of the β-cell in the offspring from malnourished mothers is still unknown and clearly requires further investigation.

## Conclusions

To date, the body of evidence indicates that maternal protein restriction during lactation programs the metabolism to reserve less energy in rat adulthood. Although several studies have shown that perinatal nutritional alterations modify both stress neuroendocrine systems, including the hypothalamus-pituitary-adrenal axis, and the sympathoadrenal system, little is known concerning the PNS in this context. However, each aspect of this system is involved in the regulation of energy metabolism. We have shown that adult male rat offspring from whose mothers were protein-restricted during lactation exhibit a low PNS activity. Rodent lactation is a crucial phase for brain development and could be considered similar to the final one-third of the human gestation period [9,87]. Although the mechanism by which protein restriction during lactation induces permanent changes in the metabolism is still largely unknown, evidence suggests that ANS changes may contribute to the impairment of glycemic homeostasis in metabolically programmed rats. Whether these modifications of the ANS will favor or impede the features of metabolic syndrome remains controversial, and this issue warrants further investigation.

## Abbreviations

ANS: Autonomic nervous system; DOHaD: Developmental origins of health and disease; PNS: Parasympathetic nervous system; K_ATP_: ATP-sensitive potassium channels; mAChRs: Cholinergic muscarinic receptors; M_3_mAChRs: Cholinergic muscarinic receptors subtype M_3_.

## Competing interests

The author declares that there are no conflicts of interest.

## Authors’ contributions

JCde O and PCFM designed, wrote and edited the manuscript; SG and CG were involved in revising critically the manuscript. All authors read and approved the final manuscript.

## Funding

This work was supported by the Brazilian Federal Foundation, the Conselho National de Desenvolvimento Científico e Tecnológico (CNPq), the Coordenação de Aperfeiçoamento de Pessoal de Nível Superior (CAPES), and the Paraná Science Foundation (Fundação Araucária).
